# Out-of-bed extubation: changing paradigms

**DOI:** 10.1186/cc10736

**Published:** 2012-03-20

**Authors:** F Dexheimer Neto, R Cremonese, J Maccari, F Carlin, C Rodrigues, A Raupp, P Vesz, C Leaes, J De Andrade

**Affiliations:** 1 Hospital Ernesto Dornelles, Porto Alegre, Brazil

## Introduction

The position of the patient at the time of extubation is an important topic as several studies have shown that early mobilization is beneficial for the critically ill patient and, generally, it occurs simultaneously with the weaning from mechanical ventilation (MV). Extubations are currently performed with the patient in a supine position (SP) with the head elevated, and there are no data available concerning the safety of removing the endotracheal tube of a patient seated in an armchair (SA). The aim of this study was to evaluate the safety of proceeding extubations in SA patients compared with those in a SP.

## Methods

A retrospective cohort study of a clinical and surgical 23-bed ICU, in a private hospital in Brazil - Hospital Ernesto Dornelles (Porto Alegre, RS, Brazil). Extubation success was the primary outcome - defined as tolerating removal of the endotracheal tube for at least 48 hours. All statistical analysis were done using SPSS version 16 and the differences between the groups were assessed using Student's *t *test and the chi-square test.

## Results

Ninety-one patients were included in the analysis - from December 2010 to June 2011. Mean (± SD) age of the population was 71 ± 12 years, mean APACHE II score was 21 ± 7.6, mean duration of MV was 2.6 ± 2 days and mean number of spontaneous breathing trials was 1.3 ± 0.6. Extubation was performed in 33 SA patients (36%) and 58 SP patients (64%), with a similar success rate of 82% and 85%, respectively (*P *>0.05). Furthermore, no significant differences between these groups were found in terms of APACHE II score, time of MV and postextubation distress or complications.

## Conclusion

The outcomes of proceeding extubation in patients seated in armchairs are similar to those extubated in supine position with the head elevated. This new practice can be considered safe and allow extubations to be performed simultaneously with early mobilization.

